# Human papillomavirus infection as a risk factor for anal and perianal skin cancer in a prospective study

**DOI:** 10.1038/sj.bjc.6600350

**Published:** 2002-07-15

**Authors:** T Bjørge, A Engeland, T Luostarinen, J Mork, R E Gislefoss, E Jellum, P Koskela, M Lehtinen, E Pukkala, SØ Thoresen, J Dillner

**Affiliations:** Department of Pathology, The Norwegian Radium Hospital, 0310 Oslo, Norway; Department of Gynecologic Oncology, The Norwegian Radium Hospital, 0310 Oslo, Norway; Division of Epidemiology, Norwegian Institute of Public Health, 0403 Oslo, Norway; Finnish Cancer Registry, Institute for Statistical and Epidemiological Cancer Research, 00170 Helsinki, Finland; Cancer Registry of Norway, Institute of Population-based Cancer Research, 0310 Oslo, Norway; Department of Oto-rhino-laryngology, The National Hospital, 0027 Oslo, Norway; Janus Committee, Norwegian Cancer Society, 0369 Oslo, Norway; National Public Health Institute, 00300 Helsinki and Oulu, Finland; Department of Medical Microbiology, Lund University, MAS University Hospital, S-20502 Malmö, Sweden

**Keywords:** human papillomavirus, anal cancer, epidemiology

## Abstract

Human papillomavirus has emerged as the leading infectious cause of cervical and other anogenital cancers. We have studied the relation between human papillomavirus infection and the subsequent risk of anal and perianal skin cancer. A case–cohort study within two large Nordic serum banks to which about 760 000 individuals had donated serum samples was performed. Subjects who developed anal and perianal skin cancer during follow up (median time of 10 years) were identified by registry linkage with the nationwide cancer registries in Finland and Norway. Twenty-eight cases and 1500 controls were analysed for the presence of IgG antibodies to HPV 16, 18, 33 or 73, and odds ratios of developing anal and perianal skin cancer were calculated. There was an increased risk of developing anal and perianal skin cancer among subjects seropositive for HPV 16 (OR=3.0; 95%CI=1.1–8.2) and HPV 18 (OR=4.4; 95%CI=1.1–17). The highest risks were seen for HPV 16 seropositive patients above the age of 45 years at serum sampling and for patients with a lag time of less than 10 years. This study provides prospective epidemiological evidence of an association between infection with HPV 16 and 18 and anal and perianal skin cancer.

*British Journal of Cancer* (2002) **87**, 61–64. doi:10.1038/sj.bjc.6600350 www.bjcancer.com

© 2002 Cancer Research UK

## 

Anal epidermoid carcinoma is a rare tumour, but its incidence has increased over the past 30 years (Goldman *et al*, 1989; Frisch *et al*, 1993; Melbye *et al*, 1994). Sexual behaviour and smoking are risk factors (Daling *et al*, 1992; Frisch *et al*, 1997). A high proportion of anal tumours are associated with human papillomavirus (HPV) infection (Melbye and Frisch, 1998). HPV 16 DNA has been reported in up to 93% of invasive squamous anal tumours and 69% of anal carcinoma *in situ* (Tilston, 1997).

Most epidemiological studies on HPV infection and anal cancer have been case–series and case–control studies using samples taken after the cancer has been diagnosed. Such studies may be subject to differential misclassification related to the presence of the disease, and provide no information on the temporal order of events. Prospective studies are generally considered crucial for causality inference. As primary prevention of HPV infection by vaccination is being evaluated, it is important to establish which cancers are likely to be amenable to prevention. Prospective seroepidemiological studies have linked HPV to vulvar, vaginal and to a subset of head and neck cancers (Bjørge *et al*, 1997a; Mork *et al*, 2001).

HPV serology, using capsids of HPV 16, 18 and 33, has been validated as a type-restricted marker of past or present HPV infection (Dillner, 1999). A serologic association of HPV 16 with incident anal cancer has been shown (Heino *et al*, 1995). In a study of the role of HPV in non-cervical anogenital cancers, we found that HPV 16 seropositivity conferred an increased risk for non-cervical anogenital cancers, but not for anal cancer (Bjørge *et al*, 1997a). This was in spite of the fact that the proportion of anal cancer cases (25%) being HPV 16 seropositive was higher than for other non-cervical anogenital cancers (24%). This difference could not be explained by the matching criteria, suggesting that random variability among the controls (*n*=59) might be an explanation.

To investigate if the negative results did imply that the association is not causal or whether it might be explained by insufficient study size, we expanded our previous study to include a larger number of controls. Further, we used a later updated linkage of our data sources for an evaluation of the risk of developing anal and perianal skin cancer following infection with HPV 16, 18, 33 and 73.

## METHODS

### Serum banks

The Finnish maternity cohort contains blood specimens collected since 1983 by the National Public Health Institute in Oulu, Finland from 460 000 pregnant women. By 1996 almost one million blood specimens had been stored. The samples are collected at maternity clinics from almost all (about 98%) pregnant women in Finland during early pregnancy (first trimester) to screen for congenital infections. The samples are stored at −20°C.

The Janus project was started in 1973 and contains about 600 000 serum samples from about 300 000 donors. The samples have been collected from people who participated in county health examinations, mostly for cardiovascular diseases, and from blood donors. The participants in the health examinations were recruited from several counties in Norway. The blood donors were from the Red Cross Blood Donor Centre in Oslo. The samples are stored at −25°C.

### Cancer registries

Both the Finnish and the Norwegian cancer registries are nationwide and population based. Since 1953 they have received notifications from hospitals, pathology and haematology laboratories, and physicians. They provide information about site, histological type, and stage of disease at the time of diagnosis. The registration of solid cancers is regarded as practically complete (Lund, 1981; Teppo *et al*, 1994). Registration was based on a modified version of International Classification of Diseases, 7th revision.

### Identification of cases and controls

The data files of the serum banks and cancer registries were linked on the basis of the personal identification number to identify anal (ICD-7 code 154.1), perianal skin (ICD-7 code 191.4) and head and neck cancers (ICD-7 codes 140–148 and 160–161). If there were several serum samples available for each case, the first sample was chosen. Five (Norway) or seven (Finland) controls were selected from the cohorts for each case. The controls were individually matched for cohort, sex, age at serum sampling (within 2 years), storage time (within 2 months), country and, in Norway, for county of residence. Neither cases nor controls had any cancer diagnoses prior to the present cancer diagnoses.

In Norway, 23 anal and perianal skin cancer cases (13 women and 10 men) were identified. Altogether 20 cases were anal cancer and three were perianal skin cancer. Fifteen of the anal cancer cases were squamous cell carcinoma, three were cloacogenic carcinoma, one was adenocarcinoma and one was transitional cell carcinoma. The three perianal skin cancers were all squamous cell carcinoma. In Finland, five cases (all anal cancer) were identified. Three cases were squamous cell carcinoma and two were adenocarcinoma. No *in situ* cases were included. Serum samples from 1500 controls were available for analysis.

In the present study, all controls were used for analysis using a case–cohort study design. The sampling with matching criteria thus only served to increase statistical power by frequency matching controls to the major cancer sites in the study. The results of the head and neck cancer analyses are published separately (Mork *et al*, 2001).

The median age at diagnosis was 57 years (range 43–68 years) for the Norwegian and 39 years (range 32–44 years) for the Finnish cases. Median time between withdrawal of serum and diagnosis was 10 years (range 1.9–22 years) and 8.3 years (range 6.0–12 years) for the Norwegian and Finnish cases, respectively.

Compared with our previous study (Bjørge *et al*, 1997a), three more anal cancer cases were identified in Norway and four in Finland. However, in Norway two of the previous anal cancer cases were excluded due to multiple malignancies. Further, two of the previous perianal skin cancer cases were also excluded, one due to multiple malignancies, the other due to histology (Paget's disease of perineum). These exclusions were decided at the outset of this study and without knowledge of the serological results of the previous study (Bjørge *et al*, 1997a).

### Laboratory methods

Seropositivity to HPV capsids was determined by an established and validated enzyme linked immunosorbent assay (ELISA) using baculovirus expressed capsids comprising both the L1 and L2 proteins, with disrupted capsids of bovine papillomavirus as control (Heino *et al*, 1995). The cut-off levels used to assign seropositivity were preassigned and, relative to internal standards, identical to the cut-offs used in previous studies (HPV 16: cut-off level 354, HPV 18, 33 and 73: cut-off level 100) (Bjørge *et al*, 1997a,b; Dillner *et al*, 1997).

All laboratory analyses were performed on masked samples.

### Statistical analyses

The data was analysed in a case–cohort design. Odds ratios (ORs) and their 95% confidence intervals (95%CI) were derived from Cox proportional hazard regression models (Cox and Oakes, 1984), using the program package Epicure (Preston *et al*, 1993; Hirosoft International Corporation, 2000). In the analyses, the ‘risk-time’ for each person, i.e. the time from serum sampling until the election date, was used as the time variable. Sex (two categories), age (three categories), time of serum sampling (four categories) and serumbank/area of residence (five categories; Finland, Oslo county, Finnmark county, other Norwegian counties and Red Cross blood donors) were adjusted for.

## RESULTS

Overall, 50% (14 patients) of the anal and perianal skin cancer cases was seropositive for HPV 16, 18, 33 or 73. Eight cases were seropositive for HPV 16, five for HPV 18, six for HPV 33 and one case was seropositive for HPV 73. There was an increased risk of developing anal and perianal skin cancer among subjects seropositive for HPV 16 (OR=3.0; 95%CI=1.1–8.2) and HPV 18 (OR=4.4; 95%CI=1.1–17), with adjustment for the other serotypes (Table 1

**Table 1:**
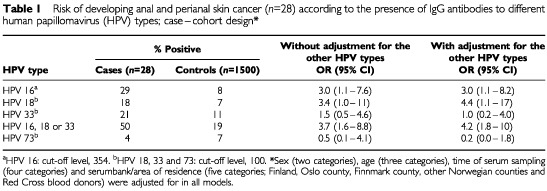
Risk of developing anal and perianal skin cancer (*n*=28) according to the presence of IgG antibodies to different human papillomavirus (HPV) types; case–cohort design*

). In the Norwegian part of the study, ORs were also calculated dichotomising on age and lag time until cancer diagnosis. Higher odds ratios for anal cancer were observed among HPV 16 seropositive subjects above 45 years of age (OR=7.3; 95%CI=1.5–37) and with less than 10 years lag (OR=6.2; 95%CI=1.4–26), with adjustment for the other serotypes. Lower odds ratios were seen for the other serotypes. No substantial differences in odds ratios were found when the analyses were restricted to anal cancer, to squamous cell carcinoma or to women, respectively (data not shown).

## DISCUSSION

The present study has provided prospective epidemiological evidence that infection with HPV 16 and 18 does confer an increased risk for future anal and perianal skin cancer. The highest risks were seen for HPV 16 seropositive patients above the age of 45 years at serum sampling and for patients with a lag time of less than 10 years.

The role of a sexually transmitted agent in the etiology of anal cancer was first suggested in 1979 (Cooper *et al*, 1979). Similar to the cervix, the anal canal has a squamocolumnar junction or transformation zone, which appears to be particularly susceptible to HPV infection, and the concomitant risk of intraepithelial neoplasia and carcinoma (Tilston, 1997). It is now well established that HPV play a central role in the pathogenesis of anogenital cancers and their precursors (International Agency for Research on Cancer, 1995; Ryan *et al*, 2000). HPV DNA has been detected in invasive as well as in anal squamous intraepithelial lesions. HPV 16 is the most common type found. *In situ* hybridisation studies have found 24–73% of cases to be HPV DNA positive, whereas the corresponding figures for Southern blot and PCR studies are 63–85% and 24–100%, respectively (International Agency for Research on Cancer, 1995). HPV DNA is almost always found integrated into the host chromosome, but it is frequently coexistent with episomal DNA in the cell nucleus (Holm *et al*, 1994; Tilston, 1997).

However, the detection of HPV DNA implies current infection only. Prior exposure is not necessarily reflected. By applying HPV serology, a marker of both past and present HPV infection, it has been possible to investigate possible temporal associations of HPV infection with anal cancer. Previously, a serologic association of HPV 16 with incident anal cancer has been reported (Heino *et al*, 1995). In our previous, prospective study, no association of HPV and anal cancer was found, although a high, but insignificant risk was found for perianal skin cancer (Bjørge *et al*, 1997a). The present expanded study is, to our knowledge, the first study providing prospective epidemiologic evidence of an association between HPV infection and anal and perianal skin cancer. Subjects seropositive for HPV 16 and also for HPV 18 were at an increased risk.

Patients with HPV DNA in their anal tumours have been reported to be about 10 years younger than those with HPV DNA-negative anal cancers (Heino *et al*, 1993). In this study, the mean age of the cases being seropositive for any HPV was 54 years, whereas the mean age of the seronegative cases was 53 years.

At present, the incidence of anal cancer is about two to three times higher in women than in men. Particularly in women, the incidence has increased substantially over the past decades. A higher proportion of anal cancer cases has been reported to be positive for HPV DNA in women compared to men (Holm *et al*, 1994; Frisch *et al*, 1997). In the present study, 28% of the female and 30% of the male cases were seropositive for HPV 16. Similar figures for HPV 18 were 17% and 20%, respectively.

In summary, this study provides prospective epidemiological evidence indicating that infection with HPV 16 and also HPV 18 does increase the risk for subsequent development of anal and perianal skin cancer.
