# Human papillomavirus infection as a risk factor for anal and perianal skin cancer in a prospective study

**DOI:** 10.1038/sj.bjc.6602116

**Published:** 2004-09-10

**Authors:** T Bjørge, A Engeland, T Luostarinen, J Mork, R E Gislefoss, E Jellum, P Koskela, M Lehtinen, E Pukkala, S Ø Thoresen, J Dillner

**Correction to:** *British Journal of Cancer* (2002) **87**, 61–64. doi:10.1038/sj.bjc.6600350

Due to an error, the database used in the above study had interchanged information on sex with those of one of the participating cohorts. Consequently, some of the results in the published article were incorrect. The authors have performed new analyses and the correct data are given below:

In a joint study of future risks for anal and perianal skin cancers in relation to baseline human papillomavirus (HPV) seropositivity nested in Norwegian and Finnish cohorts, it turns out that the information on sex had been interchanged in one of the Norwegian cohorts.

With correct information on sex, HPV 16 seropositivity conferred an increased risk of anal/perianal cancer of 3.6 (95% CI=1.3–10). Overall, 27% of the female and 31% of the male cases were seropositive for HPV 16. Similar figures for HPV 18 were 27% and 8%, respectively. By dichotomisation on age and lag in the Norwegian cohort, we found that HPV 16 seropositive men above 45 years of age had an increased risk (OR=16; 95% CI=2.6–96) as did subjects with less than 10 years lag (OR=6.9; 95% CI=1.7–27).

The corrected data are given below (Table 1

**Table 1:** Risk of developing anal and perianal skin cancer (*n*=28) according to the presence of IgG antibodies to different human papillomavirus (HPV) types; case – cohort design

	**% Positive**		**Without adjustment for the other HPV types**		**With adjustment for the other HPV types**	
**HPV type**	**Cases (*n*=28)**	**Controls (*n*=1500)**	**OR**	**95% CI**	**OR**	**95% CI**
HPV 16^a^	29	8	3.7	1.5–9.5	3.6	1.3–10
HPV 18^b^	18	7	2.9	0.9–9.2	2.9	0.8–10
HPV 33^b^	21	11	1.5	0.5–4.3	1.0	0.3–3.2
HPV 16, 18 or 33	50	19	4.0	1.7–9.4	4.5	1.9–11
HPV 73^b^	4	7	0.4	0.1–3.1	0.3	0.0–2.1

a HPV 16: cut-off level, 354.

b HPV 18, 33 and 73: cut-off level, 100.

* Sex (two categories), age (three categories), time of serum sampling (four categories) and serum bank/area of residence (five categories; Finland, Oslo county, Finnmark county, other Norwegian counties and Red Cross blood donors) were adjusted for in all models.

).

The authors apologise for any inconvenience this may have caused. For a complete list of corrections to the text of the article, please contact tone.bjorge@oslo.online.no

